# Myocarditis after COVID-19 mRNA vaccination in Norway: a nationwide validation study

**DOI:** 10.1136/openhrt-2026-004112

**Published:** 2026-05-04

**Authors:** Bendik Skinningsrud, Katarina Vlaisavljevic, Lars Sandve Oppedal, Johannes Endresen, Vilde Storesund Mohn, Kristina Fladseth, Trygve Moe Lysaker, Margareth Pleym Ribe, Thomas Friedrich Möller, Torstein Laurits Hole, Jesper Dahl, Øystein Karlstad, Hanne Løvdal Gulseth, David Benee Olsen, Margrethe Greve-Isdahl, Sara Viksmoen Watle, Mette-Elise Estensen, Kaspar Broch, Kristina H Haugaa, Nina Eide Hasselberg

**Affiliations:** 1 ProCardio Center for Innovation, Department of Cardiology, Oslo University Hospital, Rikshospitalet , Oslo, Norway; 2 Institute of Clinical Medicine, Faculty of Medicine, University of Oslo, Oslo, Norway; 3 Department of Cardiology, Haukeland University Hospital, Bergen, Norway; 4 Department of Cardiology, Nordland Hospital, Bodø, Norway; 5 Department of Cardiology, Stavanger University Hospital, Stavanger, Norway; 6 Department of Cardiology, University Hospital of North Norway, Tromsø, Norway; 7 Department of Cardiology, St. Olavs Hospital (Trondheim University Hospital), Trondheim, Norway; 8 Department of Paediatric Cardiology, Oslo University Hospital, Rikshospitalet, Oslo, Norway; 9 Department of Cardiology, Ålesund Hospital, Ålesund, Norway; 10 Norwegian Institute of Public Health, Oslo, Norway; 11 Norwegian Medical Products Agency, Oslo, Norway

**Keywords:** Myocarditis, Epidemiology, Outcome Assessment, Health Care

## Abstract

**Background:**

Myocarditis is a potentially severe adverse event after COVID-19 messenger RNA (mRNA) vaccination. Validation of reported cases is essential. We aimed to determine the occurrence, clinical characteristics and short-term outcomes of vaccine-associated myocarditis (VAM) in Norway.

**Methods:**

In this nationwide, population-based validation study, we used national health registry data and hospital electronic medical records from 27 December 2020 to 30 April 2022. We identified all Norwegian residents who received at least one dose of BNT162b2 or mRNA-1273. By cross-linking registries, we identified myocarditis within 90 days after vaccination. Diagnoses were validated through individual chart review using Brighton Collaboration criteria. VAM was defined as myocarditis without a more likely alternative cause.

**Results:**

Among 4.1 million vaccinated individuals who received 10.9 million doses, we identified 367 potential myocarditis cases. Of 349 cases reviewed, 177 (51%) were validated as VAM, corresponding to 4.5 cases per 100 000 vaccinated individuals. In total, 110 (62%) cases occurred after the second dose. Of validated cases, 139 (79%) occurred in men. Median age was 30 (IQR 24–50) years for men and 54 (IQR 32–65) years for women. Three (2%) cases were under 18 years. The median hospital stay was 4 (IQR 3–5) days, and the median ejection fraction was 55% (IQR 53%–60%). Seven (4%) patients required intensive care and two (1%) older patients died. No patient required mechanical circulatory support or heart transplantation.

**Conclusions:**

VAM occurred in 4.5 per 100 000 vaccinated individuals, based on validation of about half of registry-identified myocarditis cases. The acute clinical course was generally mild. National surveillance and systematic validation are essential for reliable estimates of vaccine-associated adverse events.

**Trial registration number:**

NCT05610423.

**WHAT IS ALREADY KNOWN ON THIS TOPIC**

- COVID-19 messenger RNA vaccination has been associated with rare cases of myocarditis.
- Most existing estimates of vaccine-associated myocarditis (VAM) are based on registry or surveillance data without systematic clinical validation.

**WHAT THIS STUDY ADDS**

- Systematic validation showed that only about half of registry-identified myocarditis cases after vaccination were true VAM, yielding an occurrence of 4.5 per 100 000 vaccinated individuals.
- Most cases occurred in young men, cardiac function was generally preserved, few required intensive care, and mortality was low.

**HOW THIS STUDY MIGHT AFFECT RESEARCH, PRACTICE OR POLICY**

- Clinical case validation supplements national surveillance systems, providing more reliable estimates of vaccine-associated adverse events to inform vaccine policy.

## Introduction

 The messenger RNA (mRNA) vaccines BNT162b2 (Comirnaty, BioNTech-Pfizer) and mRNA-1273 (Spikevax, Moderna) reduced the risk of COVID-19, hospitalisations and death, and played a crucial role in mitigating the pandemic.^1 2^ However, rare cases of vaccine-associated myocarditis (VAM) have been reported.^3^ COVID-19 mRNA-VAM was not identified in the 2020 phase III clinical trials, likely due to the rarity of the adverse event and the older age of study participants.^4 5^ The first reports emerged from Israel, where the incidence of VAM was 2.1 cases per 100 000 after at least one vaccine dose and 2.7 per 100 000 after two doses of BNT162b2.^6 7^ In large cohort studies, VAM occurred more often in younger men, particularly after the second vaccine dose, and the risk was higher with mRNA-1273 than with BNT162b2.^8 9^ Although these studies included large national cohorts and comprehensive registry data, myocarditis diagnoses were not clinically validated.

Norway launched its national COVID-19 vaccination programme in December 2020 and used mainly mRNA-based vaccines. BNT162b2 accounted for 79% of the administered doses between 2021 and April 2022.^10^ Due to reports of increased risk of VAM in younger individuals, there were concerns to expand vaccination to children and adolescents.^11^ In August 2021, Norwegian health authorities recommended BNT162b2, but not mRNA-1273, for individuals aged 12–17 years.^7^ Vaccination of children younger than 12 years was limited; only 6190 (1%) received at least one dose, primarily those with underlying risk factors. By using national registry data and systematic case validation, we aimed to determine the occurrence, clinical characteristics, and short-term outcomes of COVID-19 mRNA-VAM in Norway.

## Methods

### Study design, setting and participants

**Figure 1:**
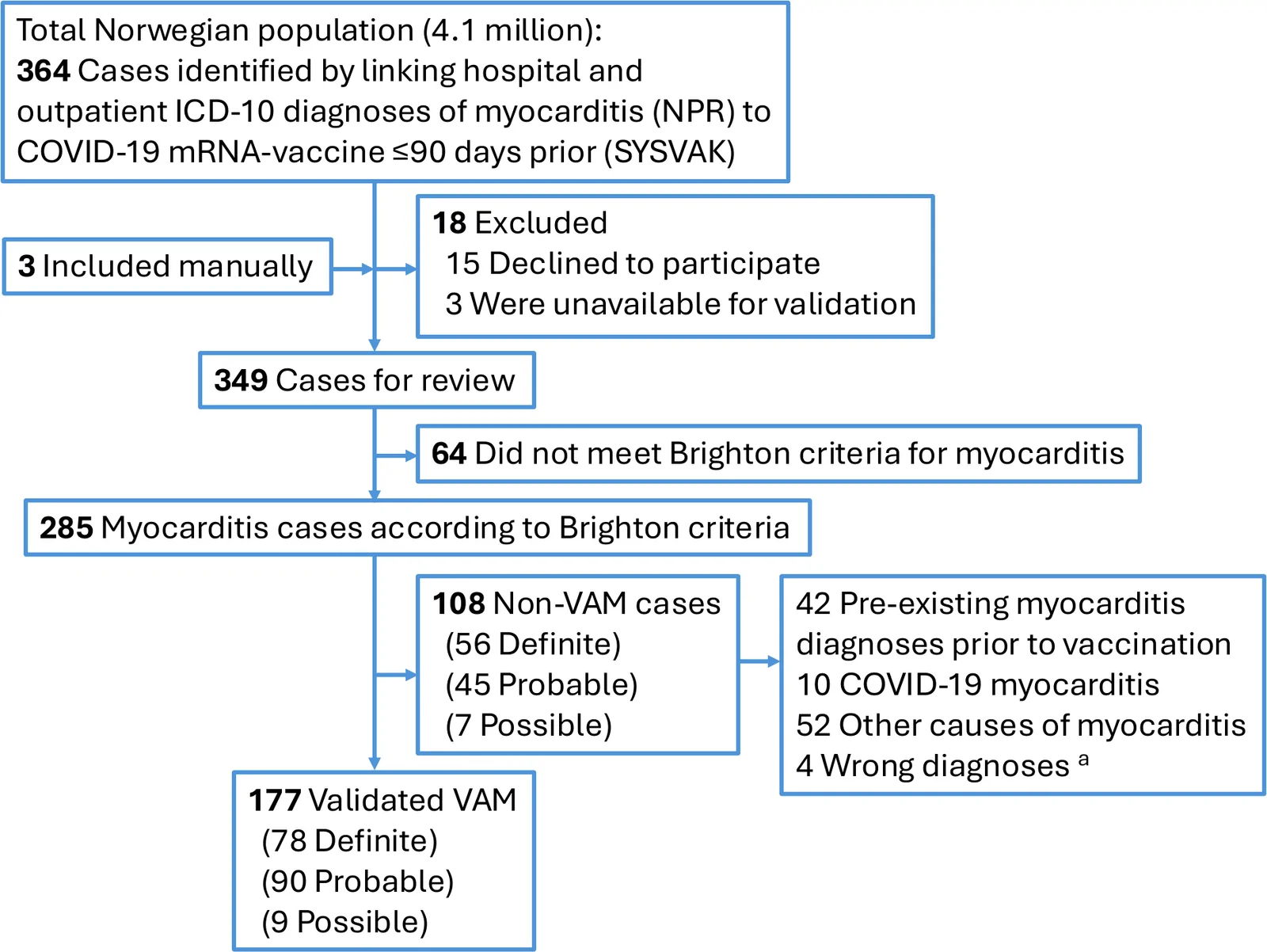
Flow chart illustrating identification of study population and validation of COVID-19 VAM. ^a^Four cases fulfilled the Brighton criteria for myocarditis; however, electronic medical record review revealed other diagnoses: apoplexy-related troponin rise (n=1), myocardial infarction with non-obstructive coronary arteries (n=1), sarcoidosis (n=1) and vaccine-associated pericarditis (n=1). ICD-10, International Statistical Classification of Diseases, 10th Revision; NPR, Norwegian Patient Registry; SYSVAK, Norwegian Immunisation Registry SYSVAK; VAM, vaccine-associated myocarditis.

This nationwide retrospective validation study included all Norwegian residents who received a COVID-19 mRNA vaccine (BNT162b2 and/or mRNA-1273) from 27 December 2020 to 30 April 2022. We used the unique personal national identity number^12^ to link national health registries and to review hospital electronic medical records (EMRs) (online supplemental methods). Individuals were eligible for case review if they had a hospital admission or outpatient contact with a diagnosis code for myocarditis (*International Statistical Classification of Diseases, 10th Revision,* online supplemental table 1) in the Norwegian Patient Registry (NPR)^13^ within 90 days of COVID-19 mRNA-vaccination registered in the Norwegian Immunisation Registry SYSVAK^14^ (figure 1). Vaccine uptake for the entire Norwegian population from 27 December 2020 to 30 April 2022 was retrieved from the emergency preparedness register for COVID-19 (Beredt C19).^10^ Patients contributed to protocol development.

### Data collection and outcome measures

We extracted patient information from hospital EMRs (online supplemental table 2) to support diagnostic adjudication and outcome assessment. The primary outcome was the estimated number of VAM per 100 000 vaccinated individuals. Additional measures were demographic and clinical characteristics, diagnostic validity, imaging and laboratory findings, treatment, and short-term outcomes of VAM (from admission to discharge). Echocardiography and cardiac magnetic resonance (CMR) imaging had been performed according to local hospital protocols in line with current recommendations.^15^

### Validation of diagnoses

Local physicians performed a structured review of EMRs to assess all potential cases of VAM identified through the registry linkage of NPR and SYSVAK. The Brighton Collaboration Criteria were applied to adjudicate diagnoses of myocarditis.^16^ We classified cases as definite, probable or possible myocarditis based on histopathology, cardiac biomarkers (troponin T/I), ECGs, symptoms, inflammatory biomarkers (C reactive protein, erythrocyte sedimentation rate) and imaging (CMR or echocardiography). Cases of myocarditis where a cause other than vaccination was deemed more plausible, including recent or ongoing SARS-CoV-2 infection, were classified as non-VAM. The remaining cases were classified as VAM. Information on recent infections and SARS-CoV-2 PCR test results at hospital admission was extracted from EMRs as part of the adjudication process.

### Statistical analysis

We used descriptive statistics to summarise demographic and clinical characteristics. Categorical variables are presented as counts and percentages, and continuous variables as medians and IQRs or as means and SD. We performed statistical comparisons using Student’s t-tests, Mann-Whitney, χ^2^ or Fisher’s exact tests, as appropriate. Missing data were not imputed, and analyses were based on available observations for each variable. In the primary analysis, we included cases of myocarditis occurring within 90 days after vaccination. In a secondary analysis, we restricted inclusion to cases within 30 days. Analyses were conducted using Stata V.18.5 (StataCorp 2023).

### Ethical considerations

The study was conducted in accordance with the Declaration of Helsinki. The requirement for active informed consent was waived by the ethics committee due to the minimal individual risk and substantial societal benefit of the study. Individuals eligible for case review through EMRs were provided with study information and given the opportunity to decline participation by denying access to their EMR for the study.

## Results

From 27 December 2020 to 30 April 2022, 4114 750 individuals in Norway received 10 915 098 COVID-19 mRNA-vaccine doses; 79% BNT162b2 and 21% mRNA-1273.^10^

### Validation of VAM

Linkage of national health registries identified 364 individuals with myocarditis diagnoses from hospital admissions or outpatient consultations occurring within 90 days after COVID-19 mRNA-vaccination. In addition, the study team was notified of three potential cases not captured in the registries. Fifteen individuals declined participation, and three cases were unavailable for validation due to technical issues with access to EMRs, leaving 349 cases for review. Of these, 285 (82%) met the Brighton Collaboration criteria for myocarditis on chart review (figure 1). We classified 177/349 cases (51%) as VAM and 108/349 (31%) as non-VAM, while 64/349 (18%) did not meet myocarditis criteria. Among the non-VAM cases, 42/108 (39%) had pre-existing myocarditis diagnoses prior to COVID-19 mRNA-vaccination, and the diagnostic code had been reiterated at follow-up after vaccination. Ten cases were adjudicated as myocarditis associated with SARS-CoV-2 infection. Four cases fulfilled the Brighton criteria but were determined on EMR review to have other final diagnoses (figure 1). When limiting registry-identified myocarditis to inpatient admissions within 30 days after vaccination as conventionally reported, 108/134 (81%) were validated as VAM, compared with 1/44 (2%) outpatient cases (online supplemental table 3).

Based on the diagnostic certainty of the 177 validated VAM cases, we classified 78 (44%) as definite, 90 (51%) as probable and 9 (5%) as possible myocarditis, in accordance with the Brighton criteria (table 1). Men accounted for 66 (85%) of definite cases, 69 (77%) of probable cases and 4 (44%) of possible cases (p=0.02). Among definite cases, 34 (44%) occurred within 7 days after vaccination, all of which were in men (median age 25 years (IQR 22–33)).

**Table 1:** Baseline characteristics of patients with VAM ≤90 days after COVID-19 vaccination (n=177)

Clinical characteristics	
Men, n (%)	139 (79)
Age, median (IQR), in women, years	54 (32–65)
<18, n (%)	1 (3)
18–29, n (%)	7 (18)
30–49, n (%)	8 (21)
50+, n (%)	22 (58)
Age, median (IQR), in men, years	30 (24–50)
<18, n (%)	2 (1)
18–29, n (%)	64 (46)
30–49, n (%)	37 (27)
50+, n (%)	36 (26)
White patients, n (%)*	159 (94)
Married or cohabiting, n (%)	68 (40)
Body mass index, median (IQR), kg/m^2^	25 (23–29)
Body surface area, mean (SD), m^2^	2.0± 0.21
**Medical history, n (%)**	
Smoker, current/previous	20 (13)/30 (19)
Prior myocarditis	5 (3)
Infection, febrile disease or COVID-19 <4 weeks prior to VAM	26 (15)
Previous cardiovascular disease	16 (9)
Hypertension	20 (11)
Diabetes mellitus	10 (6)
Autoimmune disease	22 (13)
Chronic pulmonary disease	4 (2)
Cancer	5 (3)
Other relevant medical history	17 (10)
History of cardiomyopathy among first-degree relatives	1 (1)
History of cardiomyopathy among non-first-degree relatives	2 (1)
Family history of other cardiovascular diseases	34 (33)
**Diagnostic certainty of VAM, n (%)**	
Definite	78 (44)
Probable	90 (51)
Possible	9 (5)
**Days from vaccination to VAM, median (IQR)**	22 (4–48)
**Vaccine type, n (%)**	
BNT162b2	98 (55)
mRNA-1273	79 (45)
**Vaccine dose prior to VAM**	
COVID-19 mRNA vaccine No. 1, n (%)	36 (20)
BNT162b2, n	29
mRNA-1273, n	7
COVID-19 mRNA vaccine No. 2, n (%)	110 (62)
BNT162b2, n	45
mRNA-1273, n	65
COVID-19 mRNA vaccine No. 3, n (%)	31 (18)
BNT162b2, n	24
mRNA-1273, n	7

Percentages may not total 100% due to missing data or varying response rates.

Race is not systematically registered in Norway.

* Race and ethnicity were investigator reported.

mRNA, messenger RNA; VAM, vaccine-associated myocarditis.

### Baseline characteristics and occurrence

**Figure 2:**
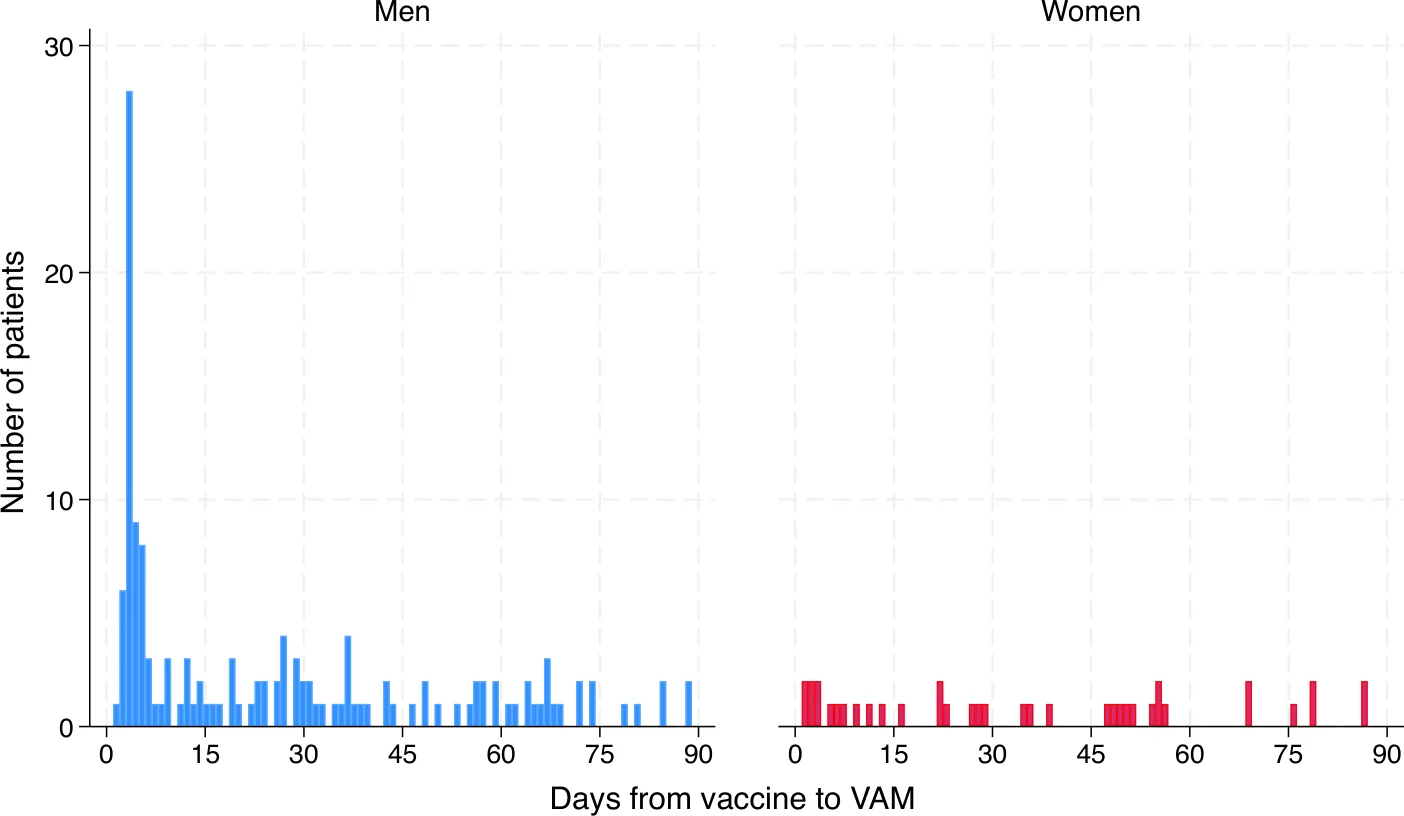
Days from last receipt of COVID-19 vaccine dose to onset of validated VAM. VAM, vaccine-associated myocarditis.

The estimated occurrence of VAM was 4.5 cases per 100 000 vaccinated individuals, calculated by applying the validation rate from chart-reviewed cases (51%) to the total number of identified cases (n=367). Of the 177 validated VAM cases, 139 (79%) occurred in men (p<0.001 for difference between sexes). The median age was 30 (IQR 24–50) years in men and 54 (IQR 32–65) years in women (p<0.001). Only three cases were identified in individuals younger than 18 years (table 1). A subgroup analysis of patients with VAM aged <30 years is presented in online supplemental table 4. Among the 177 validated cases, 109 (62%) occurred within 30 days after vaccination (online supplemental table 5). Symptom onset peaked 3 days after vaccination (figure 2). The median time to onset was 22 (IQR 4–48) days for cases occurring within 90 days after vaccination and 5 (IQR 3–16) days for those within 30 days. Among patients with available exercise data, more than half reported recent physical activity, including moderate to intense exertion (online supplemental table 6).

### Vaccine type and dose interval

Median interval between the first and second doses among cases in which VAM occurred after dose 2 (n=110) was 42 (IQR 35–56) days, and the median interval between the second and third doses among cases with VAM after dose 3 (n=31) was 174 (IQR 146–202) days. Median age was 29 (IQR 23–52) years among cases occurring after dose 2 and 53 (IQR 35–68) years after dose 3 (p<0.001).

### Clinical data

Chest pain was the most common presenting symptom, followed by shortness of breath and fever (table 2). Two patients were managed as outpatients, whereas 175 patients were hospitalised. The median duration of hospitalisation was 4 (IQR 3–5; range, 1–49) days. Core clinical data, including vital signs, ECG findings and laboratory markers are summarised in table 2. Corresponding data for the subgroup of patients with VAM within 30 days after vaccination are presented in online supplemental table 7. Median troponin T and troponin I levels are shown in online supplemental figure 1. Two patients tested positive for SARS-CoV-2 by PCR at hospital admission (table 2). Based on the temporal relationship between vaccination and symptom onset, clinical presentation, and absence of systemic COVID-19 illness, these cases were adjudicated as VAM rather than SARS-CoV-2-associated myocarditis. One of these patients underwent endomyocardial biopsy. Additional clinical data are provided in online supplemental table 8.

**Table 2:** Symptoms and clinical data in patients with VAM ≤90 days after COVID-19 vaccination (n=177)

Presenting symptoms, n (%)		
Acute chest pain	143 (81)	
Shortness of breath	56 (32)	
Fever	49 (28)	
Fatigue	24 (14)	
Myalgia/arthralgia	22 (12)	
Nausea/emesis	18 (10)	
Shoulder and/or upper back pain	17 (10)	
Palpitations	16 (9)	
Diaphoresis	15 (8)	
Syncope	5 (3)	
**Clinical parameters**	**Admission**	**Discharge**
	n=164	n=63
Systolic blood pressure, mm Hg	131±18	117 (108–128)
Diastolic blood pressure, mm Hg	79±12	71±12
Heart rate, beats/min	80±18	69±12
Oxygen saturation, %	98 (97–100)	98 (97–99)
**ECG**	n=156	n=43
Sinus rhythm, n (%)	152 (97)	42 (98)
Atrial fibrillation, n (%)	3 (2)	1 (2)
Premature atrial contractions, n (%)		
PVC/VES, n (%)	5 (3)	2 (5)
PR interval, ms	152 (136–175)	150±22
QRS-duration, ms	94 (87–102)	93±9
**Biochemical parameters**	n=157	n=120
Haemoglobin, g/dL	14.3 (13.3–15.0)	13.9±1.3
Creatinine, µmol/L	76 (68–87)	81 (71–94)
N-terminal pro-B-type natriuretic peptide, ng/L	300 (98–914)	158 (123–271)
C reactive protein, mg/L	26 (5–61)	12 (5–30)
Erythrocyte sedimentation rate, mm/hour	16 (7–23)	11 (8–26)
Positive SARS-CoV-2 PCR test, n (%)	2 (1)	

Results are reported as median (IQR), n (%) or mean (SD).

PVC/VES, premature ventricular contraction/ventricular extrasystole; VAM, vaccine-associated myocarditis.

### Imaging findings

The mean left ventricular (LV) end-diastolic diameter was 50±5 mm, and the median ejection fraction was 55% (IQR 53%–60%) (table 3). Analyses of patients with VAM within 30 days after vaccination (online supplemental table 9) did not differ substantially from those of the total cohort. Additional echocardiographic data for both cohorts, including data at discharge, are presented in online supplemental table 10. Pericardial or pleural effusion or pericardial inflammation detected by chest X-ray, chest CT, echocardiography or CMR was identified in 17/165 (10%) patients at admission, 29/161 (18%) during hospitalisation and 24/156 (15%) at discharge.

**Table 3:** Imaging studies at admission of patients with VAM ≤90 days after COVID-19 vaccination

Chest X-ray, n (%)	n=108
Pleural effusion	5 (5)
Infiltration	5 (5)
Cardiomegaly	1 (1)
Other findings*	6 (6)
**Chest CT, n (%)**	**n=34**
Pulmonary embolus	0
Pneumonia	1 (3)
Pleural effusion	2 (6)
Pericardial effusion	
Other findings†	6 (18)
**Coronary angiography, n (%)**	**n=92**
CT coronary angiography	43 (47)
Invasive coronary angiography	49 (53)
Atherosclerosis	15 (16)
Stenosis	2 (2)
Calcification	
Percutaneous intervention‡	1 (1)
**Echocardiography**	**n=72**
LV ejection fraction, median (IQR), %	55 (53–60)
LV end diastolic diameter, mean (SD), mm	50±5
LV end systolic diameter, mean (SD), mm	33±5
LV end diastolic volume, mean (SD), ml/m^2^	129±26
Interventricular septum diastolic diameter, median (IQR), mm	9 (8 – 11)
Left atrial volume indexed, mean (SD), ml/m^2^	25±11
Global longitudinal strain, mean (SD), %	−18.1±3.8
**Cardiac magnetic resonance**	**n=114**
LV ejection fraction, mean (SD), %	58±9
LV end diastolic volume index, mean (SD), ml/m^2^	84±18
LV mass index, mean (SD), g/m^2^	65±18
Findings consistent with myocarditis, n/N (%) §	50/70 (71)

Percentages may not total 100% due to missing data or varying response rates.

* Includes atelectasis (n=1), pulmonary congestion (n=4) and pulmonary sarcoidosis (n=1).

† Includes bilateral lung consolidations without features of infection (n=3), emphysema (n=1) and enlarged lymph nodes (n=2).

‡ Optical coherence tomography-guided balloon dilatation without stent placement.

§ Defined according to the 2018 revised Lake Louise Criteria, requiring the presence of both T2-based markers of oedema and T1-based markers of non-ischaemic injury. Of the 114 patients with CMR, 70 had both T2- and T1-based sequences acquired and were therefore eligible for full Lake Louise assessment. The percentage is calculated from this subset.

LV, left ventricular; VAM, vaccine-associated myocarditis.

### Treatment and outcomes

Among 170 VAM cases with available treatment data, 68 (40%) patients received colchicine and/or non-steroidal anti-inflammatory drugs. Thirty-two (19%) patients received β-blockers and 15 (9%) were treated with heart failure medications (online supplemental table 11). Seven (4%) patients required treatment in the intensive care unit (ICU), of whom five were men younger than 30 years. Among these five men, onset of VAM occurred within 7 days (n=2), 30 days (n=2) or 90 days (n=1) after vaccination. ICU stays ranged from 1 day to 5 days among the five men, and none required respiratory support.

The remaining two ICU patients were older than 65 years, had significant comorbidities and reduced LV function during hospitalisation. Both patients required prolonged ICU care and died during follow-up. One had biopsy-confirmed definite myocarditis presenting more than 2 months after vaccination, and the other developed probable myocarditis within 30 days and required invasive ventilation. Given the advanced age, comorbidities and delayed symptom onset, a causal relationship between vaccination and death could not be established. No patients received mechanical circulatory support or heart transplantation.

### Children and adolescents

A total of 11 individuals younger than 18 years were identified through registry linkage, of whom 9 were available for validation. Validation confirmed VAM in three adolescents aged 16–17 years (two male and one female). One case was classified as definite VAM and two as probable VAM. Symptom onset occurred 3–35 days after the first or second dose of BNT162b2. One patient was managed as an outpatient, and the other two required hospitalisation, including one with reduced LV function. No validated VAM was identified among children aged 5–15 years. In two younger children, parental consent for validation was not obtained.

## Discussion

In this nationwide validation study including all 4.1 million vaccinated individuals in Norway, 51% of registry-identified myocarditis cases within 90 days of vaccination were validated as VAM, corresponding to an estimated 4.5 cases per 100 000 vaccinated individuals. The occurrence was highest among men aged 18–29 years, particularly after the second vaccine dose and within 30 days after vaccination. Symptom onset peaked at day 3 after vaccination. Female patients with VAM had a median age of 54 years compared with 30 years in men and tended to present later after vaccination. Most patients had preserved cardiac function at presentation and throughout hospitalisation, and the clinical course was generally mild.

Our thorough review of patient medical records and individual validation of diagnoses may have contributed to a lower case count compared with studies strictly based on register data.^17^

Given the comprehensive Norwegian health registries, most true VAM cases were likely captured. Furthermore, diagnostic codes for myocarditis have shown an 85% positive predictive value among patients younger than 60 years,^18^ consistent with the 82% (285/349) of registry-identified cases in our study validated as myocarditis on chart review.

VAM predominantly affected young men shortly after the second dose, consistent with earlier findings.^9 19^ Hormonal and immunological factors may contribute to the greater susceptibility in men.^20^ Interestingly, our data also suggest that VAM occurred more frequently in women over the age of 50 years than in younger women, highlighting that cardiac inflammation after vaccination is not limited to young men.^6 7^ These subgroup differences underscore the need for further research into VAM risk profiles and pathophysiology.

The definition of VAM in this study was based on temporal association with vaccination and exclusion of more probable causes of myocarditis, although this does not establish causality. To minimise underestimation and missing cases, we applied a time frame of 90 days, which may have increased inclusion of unrelated cases. A diagnosis of definite myocarditis according to Brighton Collaboration criteria was more common within 30 days of vaccination. This observation is consistent with expected lower diagnostic certainty with the weaker temporal association with vaccination beyond 30 days. In contrast, women were more frequently classified as possible myocarditis and tended to present later after vaccination. These findings may reflect sex differences in clinical presentation or timing of VAM. However, the findings should be interpreted cautiously given the limited number of cases.

Established risk factors for VAM include age, sex, vaccine type, dose number, and dose interval.^19 21^ The risk of VAM was higher following vaccination with mRNA-1273 compared with BNT162b2, particularly in men and after the second dose. This aligns with previously reported excess risks of 9–28 vs 4–7 cases per 100 000 vaccinated individuals within 28 days after a second dose of mRNA-1273 and BNT162b2, respectively.^9^ Prior studies suggest that shorter intervals between doses increase the risk of myocarditis while extended intervals reduce the risk up to fourfold without compromising vaccine effectiveness.^21 22^ Norway’s early extension of dosing intervals for adults younger than 45 years, introduced for logistical rather than safety reasons, may have contributed to a lower risk.^21^

The level of physical activity reported in our study is common among young adults and not a proven risk factor. However, vigorous exercise during systemic inflammation may influence immune or inflammatory responses and affect disease course in susceptible individuals.^23^ The relationship between exercise, immune modulation, and myocardial inflammation warrants further investigation in vaccine-associated cases.

Although our findings align with international data showing lower myocarditis rates in adolescents than in adults aged 18–29 years, the near absence of VAM among Norwegian adolescents aged 16–17 years—most of whom received two vaccine doses with an extended interval—suggests that the occurrence may be even lower than previously reported, particularly compared with US surveillance data.^24 25^ The mild clinical course observed in this age group is consistent with prior studies.^24^ The low number of cases overall likely reflects Norway’s early recommendation not to administer mRNA-1273 in individuals younger than 18 years and to use a single-dose schedule for adolescents aged 12–15 years, while vaccination of younger children remained limited.^26 27^

Our findings add to the growing body of evidence that VAM after COVID-19 mRNA vaccination is generally associated with a favourable short-term prognosis.^17 28^ Most patients were managed conservatively, and intensive care was rarely required. Nevertheless, severe presentations were observed, consistent with prior reports^17 28 29^ suggesting that outcomes such as fulminant myocarditis and death, although uncommon, are not absent. Hence, caution should be warranted in extrapolating the overall favourable prognosis of VAM to all patient subgroups. The two deaths in our cohort occurred several weeks after vaccination in older individuals with significant comorbidities and in-hospital complications. These findings align with prior reports suggesting that VAM is typically less severe than viral or COVID-19-related myocarditis.^17 29^ In a nationwide Korean validation study, however, severe myocarditis occurred in 20% of cases and the fatality rate was 4.4%.^30^ Possible explanations include restriction of case identification to 42 days after vaccination, inclusion of non-mRNA vaccines (ChAdOx1 and Ad26.COV2.S), potential genetic or population differences or random variation. Long-term follow-up studies and meta-analyses suggest that postvaccine myocarditis generally has a more favourable prognosis than myocarditis following SARS-CoV-2 infection or conventional viral aetiologies. Most patients with postvaccine myocarditis have resolution of symptoms and normalisation of cardiac function within weeks and low rates of rehospitalisation, cardiovascular events and mortality.^17 29 31 32^

An important strength of this study is the linkage of national health registries, which enabled identification of all hospital-registered myocarditis cases in the entire vaccinated Norwegian population. Nationwide access to all hospital records allowed detailed case validation. Many outpatient consultations initially identified in the registries were found on validation to represent follow-up of prior myocarditis rather than new-onset myocarditis due to the vaccine, underscoring the importance of diagnostic validation. Applying the Brighton Collaboration criteria further strengthened case classification, enhancing reliability for future comparisons and meta-analyses. Finally, by analysing cases within both 90-day and 30-day postvaccination periods, we balanced comprehensive case capture with focused temporal assessment. The 30-day period appeared more clinically meaningful, showing higher diagnostic certainty and stronger temporal association with vaccination.

### Limitations

Despite the comprehensiveness of the Norwegian registries and the thorough individual case validation, our study has important limitations. The registries capture only individuals evaluated in hospitals or outpatient clinics; consequently, any mild cases of VAM in persons who did not seek medical attention were missed. Myocarditis cases without a myocarditis diagnosis code at discharge would likewise not have been captured in the registry data, leading to an underestimation of the true occurrence. A few individuals identified through the registry linkage did not permit validation, and their risk characteristics may have differed from validated cases, potentially introducing bias. The Brighton Collaboration criteria may exclude mild or atypical presentations, and incomplete documentation in EMRs could have led to missed diagnoses. In addition, imaging modalities and protocols varied between hospitals, reflecting real-world differences in clinical practice during the pandemic.

VAM classification was based on temporal proximity to vaccination and on identifying cases in which no alternative cause was more likely than vaccination. Cases within 30 days after vaccination are more likely to represent true VAM than those occurring up to 90 days, as alternative causes become more probable over time. Most epidemiological studies report symptom onset within 3–7 days,^8 9^ whereas broader definitions—such as Brighton’s 6-week window^33^ or >120 days in passive surveillance^34^—may include less causally related cases. Harmonised definitions are needed to improve attribution and comparability across studies. Notably, the study data set was not linked to the Norwegian Surveillance System for Communicable Diseases, and individual-level data on SARS-CoV-2 infections occurring after vaccination were therefore unavailable. Consequently, we cannot determine how many VAM cases subsequently contracted COVID-19, nor exclude SARS-CoV-2 infection as a contributing factor to myocarditis in individual cases. Future studies linking vaccine surveillance data with individual infection registries would strengthen the ability to distinguish VAM from infection-associated myocarditis.

Finally, clinical follow-up data were limited to the index hospitalisation, and the present study therefore provides no information on long-term cardiac sequelae such as persistent or recurrent myocarditis, myocardial dysfunction or arrhythmias.

## Conclusions

This nationwide validation study showed that among all 4.1 million individuals vaccinated with COVID-19 mRNA vaccines in Norway, VAM occurred in 4.5 per 100 000 individuals, predominantly in young men shortly after the second vaccine dose. Female patients were older and tended to present later with lower diagnostic certainty. In adolescents and children, VAM was almost absent. Most VAM cases showed preserved cardiac function and favourable short-term outcomes. The findings underscore the value of combining comprehensive registry data with rigorous clinical validation to provide accurate estimates of VAM occurrence and severity, and to strengthen vaccine safety surveillance.

## Supplementary material

10.1136/openhrt-2026-004112 online supplemental figure 1

10.1136/openhrt-2026-004112 online supplemental file 1

10.1136/openhrt-2026-004112 online supplemental file 2

## Data Availability

Data are available upon reasonable request.
