# Supplementary figures and images for: Myocarditis after COVID-19 mRNA vaccination in Norway: a nationwide validation study
Source: Open Heart. 2026 May 4;13(1):e004112. doi: 10.1136/openhrt-2026-004112 (PMC13141113; doi:10.1136/openhrt-2026-004112)

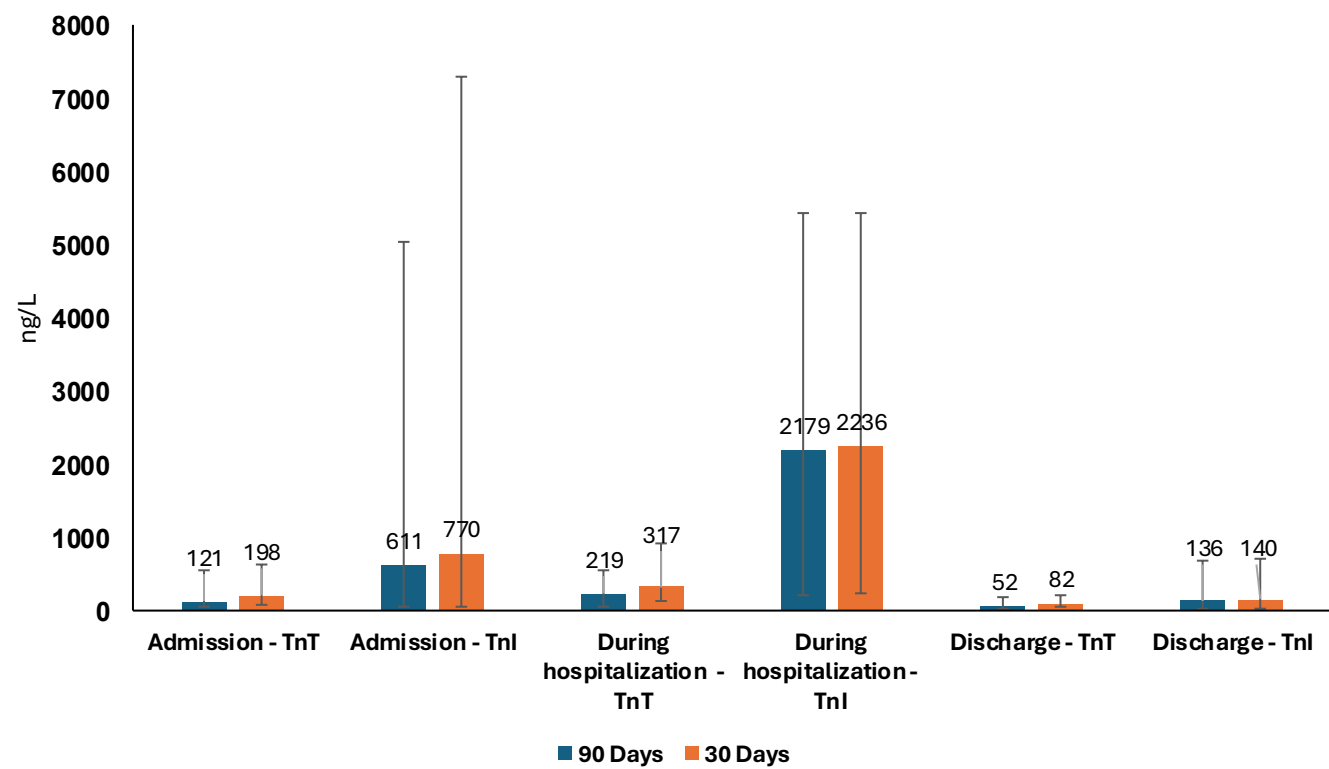

Supplement: online supplemental figure 1 [file openhrt-13-1-s001.pdf]
