# Supplementary material for: Myocarditis after COVID-19 mRNA vaccination in Norway: a nationwide validation study
Source: Open Heart. 2026 May 4;13(1):e004112. doi: 10.1136/openhrt-2026-004112 (PMC13141113; doi:10.1136/openhrt-2026-004112)
Supplement: online supplemental file 1 [file openhrt-13-1-s002.pdf]

Supplemental Material

Graphical Abstract Text

This nationwide validation study included all 4.1 million individuals vaccinated with COVID-19 mRNA vaccines in Norway. Myocarditis cases within 90 days of vaccination were identified through registry linkage and validated by chart review using Brighton criteria. Of 349 reviewed cases, 177 (51%) were validated as vaccine-associated myocarditis (VAM), corresponding to 4.5 per 100 000 vaccinated individuals. Most cases occurred in young men, cardiac function was generally preserved, few required intensive care, and mortality was low.

Supplemental Methods. Description of data sources in Norway

Supplemental Table 1. Myocarditis diagnostic codes from ICD-10

Supplemental Table 2. Participating hospitals

Supplemental Table 3. Validation of inpatient versus outpatient myocarditis after COVID-19 mRNA-vaccination

Supplemental Table 4. Baseline characteristics of patients aged <30 years with vaccine-associated myocarditis

Supplemental Table 5. Baseline characteristics of patients with vaccine-associated myocarditis ≤30 days after COVID-19 vaccination

Supplemental Table 6. Physical activity between vaccination and admission in patients with vaccine-associated myocarditis

Supplemental Table 7. Symptoms and clinical data in patients with vaccine-associated myocarditis ≤30 days after COVID-19 vaccination

Supplemental Table 8. Additional clinical data in patients with vaccine-associated myocarditis

Supplemental Table 9. Imaging studies at admission of patients with vaccine-associated myocarditis ≤30 days after COVID-19 vaccination

Supplemental Table 10. Additional echocardiographic data at admission and discharge in patients with vaccine-associated myocarditis

Supplemental Table 11. Treatment of patients with vaccine-associated myocarditis

Supplemental References

SUPPLEMENTAL METHODS

Description of data sources in Norway

The Norwegian national health registries in this study contain registrations of all contacts with health care services, which are mandatory to report, and linked to reimbursement.[1] Diagnostic codes and dates of contact are registered for each individual with a personal identification number (pin), which is issued to all citizens in Norway at birth or immigration for identification and administrative purposes. The pin enables linkages of individual level information across registries. For this study, data were obtained from the following sources:

Norwegian Immunisation Registry

The Norwegian Immunisation Registry SYSVAK is a register of vaccines administered in Norwegian vaccination programs, with mandatory registration of all COVID-19 vaccinations.[2] The information includes a personal identifier and the date of vaccination. Vaccine products are specified by a unique identifier.

Norwegian Patient Registry

The Norwegian Patient Registry (NPR) contains individual-level information on all contacts with specialist healthcare services.[3] Registered information includes admission and discharge dates, and diagnostic codes during hospital stays or outpatient visits. These codes follow the International Classification of Diseases, Tenth Revision (ICD-10). We used these codes to identify hospitalisations for myocarditis.

Emergency preparedness register for COVID-19 (Beredt C19)

The Emergency preparedness register for COVID-19 (Beredt C19) was established according to the Health preparedness act §2-4 to assess risk and implement measures during the COVID-19 pandemic. Beredt C19 received registry data from mandatory Norwegian health registries and other relevant data sources at varying time intervals, commonly on a weekly basis.

For the current study, data from SYSVAK and the National Population Register was used to describe vaccine uptake for the general population.

Supplemental Table 1. Myocarditis diagnostic codes from ICD-10

ICD-10 code	Diagnosis
I40.0	Infective myocarditis
I40.1	Isolated myocarditis
I40.8	Other acute myocarditis
I40.9	Acute myocarditis, unspecified
I41.1	Myocarditis in viral diseases classified elsewhere
I41.8	Myocarditis in other diseases that are classified elsewhere
I51.4	Myocarditis, unspecified

Abbreviations: ICD-10, International Statistical Classification of Diseases, Tenth Revision.

Supplemental Table 2. Participating hospitals

Health region	Hospitals
South-Eastern Norway Regional Health Authority	Oslo University Hospital, Akershus University Hospital, Vestfold Hospital Trust, Innlandet Hospital Trust (Hamar, Gjøvik, Lillehammer, Elverum), Telemark Hospital Trust (Skien, Notodden), Østfold Hospital Trust – Kalnes, Southern Norway Regional Hospital, Vestre Viken Hospital Trust (Drammen, Kongsberg, Ringerike, Bærum), Diakonhjemmet Hospital, Lovisenberg Diaconal Hospital
Central Norway Regional Health Authority	Møre and Romsdal Hospital Trust (Ålesund, Kristiansund, Molde, Volda), St. Olavs Hospital (Øya, Orkdal, Røros), Nord-Trøndelag Hospital Trust (Levanger, Namsos)
Western Norway Regional Health Authority	Stavanger University Hospital, Fonna Hospital Trust (Stord and Haugesund, Odda), Haukeland University Hospital, Førde Hospital Trust (Lærdal, Nordfjord, Førde), Haraldsplass Deaconess Hospital
Northern Norway Regional Health Authority	Finnmark Hospital Trust (Kirkenes, Alta, Hammerfest), Nordland Hospital Trust (Bodø), University Hospital of North Norway, Helgeland Hospital Trust

Norwegian hospitals covering all individuals in Norway contributed to the study.

Supplemental Table 3. Validation of inpatient versus outpatient myocarditis after COVID-19 mRNA-vaccination

	Cases for review, n	Validated VAM, n (%)
**Within 90 days after vaccination**	349	177 (51)
Outpatients	90	2 (2)
Inpatient admissions	259	175 (68)
**Within 30 days after vaccination**	178	109 (61)
Outpatients	44	1 (2)
Inpatient admissions	134	108 (81)

Abbreviations: VAM, vaccine-associated myocarditis.

Supplemental Table 4. Baseline characteristics of patients aged <30 years with vaccine-associated myocarditis

	VAM ≤90 days after vaccination (n=74)	VAM ≤30 days after vaccination (n=51)
**Clinical characteristics**		
Men, n (%)	66 (89)	47 (92)
White patients, n (%) [a]	69 (97)	47 (98)
Married or cohabiting, n (%)	8 (11)	5 (10)
Body mass index, median (IQR), kg/m^2	24 (21 – 28)	23 (21 – 28)
Body surface area, mean (SD), m^2	2.0±0.21	2.0±0.18
**Medical history, n (%)**		
Smoker, current/ previous	7 (9)/ 2 (2)	5 (10)/ 0
Prior myocarditis	1 (1)	1 (2)
Infection, febrile disease or COVID-19 <4 weeks prior to VAM	2 (3)	0
Previous cardiovascular disease		
Diabetes mellitus	2 (3)	2 (4)
Autoimmune disease	6 (8)	4 (8)
Chronic pulmonary disease	1 (1)	0
Other relevant medical history	4 (6)	1 (2)
History of cardiomyopathy among first-degree relatives		
History of cardiomyopathy among non-first-degree relatives	1 (1)	0
Family history of other cardiovascular diseases	13 (18)	7 (15)
**Diagnostic certainty of VAM, n (%)**		
Definite	32 (43)	25 (49)
Probable	38 (51)	24 (47)
Possible	4 (5)	2 (4)
**Days from vaccination to VAM, median (IQR)**	5 (3 – 36)	3 (3 – 5)
**Vaccine type, n (%)**		
BNT162b2	25 (34)	12 (24)
mRNA-1273	49 (66)	39 (76)
**Vaccine dose prior to VAM**		
COVID-19 mRNA vaccine No. 1, n (%)	13 (18)	6 (12)
BNT162b2, n	9	4
mRNA-1273, n	4	2
COVID-19 mRNA vaccine No. 2, n (%)	57 (77)	44 (86)
BNT162b2, n	12	7
mRNA-1273, n	45	37
COVID-19 mRNA vaccine No. 3, n (%)	4 (5)	1 (2)
BNT162b2, n	4	1
mRNA-1273, n	0	0

Percentages may not total 100% due to missing data or varying response rates. Abbreviations: VAM, vaccine-associated myocarditis.

[a] Race and ethnicity were investigator reported. Race is not systematically recorded in Norway.

Supplemental Table 5. Baseline characteristics of patients with vaccine-associated myocarditis ≤30 days after COVID-19 vaccination (n=109)

**Clinical characteristics**	
Men, n (%)	90 (83)
Age, median (IQR), in women, years	47 (30 – 69)
<18, n (%)	0
18 – 29, n (%)	4 (21)
30 – 49, n (%)	6 (32)
50+, n (%)	9 (47)
Age, median (IQR), in men, years	29 (23 – 44)
<18, n (%)	2 (2)
18 – 29, n (%)	45 (50)
30 – 49, n (%)	25 (28)
50+, n (%)	18 (20)
White patients, n (%) [a]	98 (95)
Married or cohabiting, n (%)	38 (37)
Body mass index, median (IQR), kg/m^2	25 (23 – 28)
Body surface area, mean (SD), m^2	2.0±0.21
**Medical history, n (%)**	
Smoker, current/ previous	8 (8)/ 15 (18)
Prior myocarditis	4 (4)
Infection, febrile disease or COVID-19 <4 weeks prior to VAM	14 (14)
Previous cardiovascular disease	6 (6)
Hypertension	9 (9)
Diabetes mellitus	6 (6)
Autoimmune disease	10 (10)
Chronic pulmonary disease	2 (2)
Cancer	2 (2)
Other relevant medical history	6 (6)
History of cardiomyopathy among first-degree relatives	0
History of cardiomyopathy among non-first-degree relatives	1 (1)
Family history of other cardiovascular diseases	20 (20)
**Diagnostic certainty of VAM, n (%)**	
Definite	51 (47)
Probable	54 (50)
Possible	4 (4)
**Days from vaccination to VAM, median (IQR)**	5 (3 – 16)
**Vaccine type, n (%)**	
BNT162b2	49 (45)
mRNA-1273	60 (55)
**Vaccine dose prior to VAM**	
COVID-19 mRNA vaccine No. 1, n (%)	21 (19)
BNT162b2, n	16
mRNA-1273, n	5
COVID-19 mRNA vaccine No. 2, n (%)	73 (67)
BNT162b2, n	20
mRNA-1273, n	53
COVID-19 mRNA vaccine No. 3, n (%)	15 (14)
BNT162b2, n	13
mRNA-1273, n	2

Percentages may not total 100% due to missing data or varying response rates. Abbreviations: VAM, vaccine-associated myocarditis.

[a] Race and ethnicity were investigator reported. Race is not systematically recorded in Norway.

Supplemental Table 6. Physical activity between vaccination and admission in patients with vaccine-associated myocarditis

<table>
<tr><th></th><th>VAM ≤90 days after vaccination (n=31)</th><th>VAM ≤30 days after vaccination (n=15)</th></tr>
<tr><td>Engaged in physical activity, n (%)</td><td>18 (58)</td><td>10 (67)</td></tr>
<tr><td>Types of activity, n</td><td>n=16</td><td>n=8</td></tr>
<tr><td>Aerobics</td><td>9</td><td>6</td></tr>
<tr><td>Weightlifting</td><td rowspan="2">2</td><td>0</td></tr>
<tr><td>Walking/ yoga</td><td rowspan="2">1</td></tr>
<tr><td>Military training/ rowing/ swimming</td><td>3</td></tr>
<tr><td>Exercise intensity, n</td><td>n=18</td><td>n=10</td></tr>
<tr><td>Intense</td><td>5</td><td rowspan="3">2</td></tr>
<tr><td>Moderate</td><td>4</td></tr>
<tr><td>Mild</td><td>2</td></tr>
<tr><td>Unknown</td><td>7</td><td>4</td></tr>
<tr><td>Exercise duration, n</td><td>n=4</td><td>n=2</td></tr>
<tr><td>>5 hours per week</td><td>1</td><td>0</td></tr>
<tr><td>2.5-5 hours per week</td><td>2</td><td rowspan="2">1</td></tr>
<tr><td><2.5 hours per week</td><td>1</td></tr>
</table>

Abbreviations: VAM, vaccine-associated myocarditis.

Supplemental Table 7. Symptoms and clinical data in patients with vaccine-associated myocarditis ≤30 days after COVID-19 vaccination (n=109)

**Presenting symptoms, n (%)**		
Acute chest pain	90 (83)	
Shortness of breath	35 (32)	
Fever	30 (28)	
Myalgia/arthralgia	18 (17)	
Fatigue	13 (12)	
Shoulder and/or upper back pain	12 (11)	
Nausea/emesis	11 (10)	
Diaphoresis	11 (10)	
Palpitations	9 (8)	
Syncope	4 (4)	
**Clinical parameters**	**Admission**	**Discharge**
	n=100	n=39
Systolic blood pressure, mm Hg	131 (117 – 140)	119±16
Diastolic blood pressure, mm Hg	80±12	70±12
Heart rate, beats/min	81±17	69±12
Oxygen saturation, %	98 (97 – 100)	98±1
**Electrocardiogram**	n=95	n=28
Sinus rhythm, n (%)	92 (97)	27 (96)
Atrial fibrillation, n (%)<br>Premature atrial contractions, n (%)	3 (3)	1 (4)
PVC/VES, n (%)	2 (2)	0
PR interval, ms	150 (136 – 168)	150±17
QRS-duration, ms	92 (86 – 101)	93±9
**Biochemical parameters**	n=94	n=74
Haemoglobin, g/dL	14.3±1.1	14.5 (13.4 – 15.1)
Creatinine, µmol/L	77 (68 – 85)	81 (71 – 92)
N-terminal pro-B-type natriuretic peptide, ng/L	300 (141 – 665)	155 (117 – 222)
C-reactive protein, mg/L	31 (8 – 57)	10 (5 – 30)
Erythrocyte sedimentation rate, mm/hour	16 (7 – 22)	9 (7 – 51)

Results are reported as median (IQR), n (%), or mean (SD). Abbreviations: PVC/VES, premature ventricular contraction/ventricular extrasystole; VAM, vaccine-associated myocarditis.

Supplemental Table 8. Additional clinical data in patients with vaccine-associated myocarditis

Clinical parameters	VAM within 90 days after vaccination		VAM within 30 days after vaccination	
	**Admission** n=164	**Discharge** n=63	**Admission** n=100	**Discharge** n=39
Temperature, °C	36.8 (36.5 – 37.2)	36.5±0.5	36.7 (36.4 – 37.1)	36.5±0.5
Pericardial friction rub, n (%)	1 (1)			
Distant heart sounds, n (%)	2 (1)	0	0	0
Pulsus paradoxus, n (%)	0			
Systolic murmur, n (%)	3 (2)		1 (1)	
Respiratory rate, breaths/min, n (%)	17 (15 – 20)	16±2	17 (15 – 20)	15±2
Peripheral oedema, n (%)	3 (2)	0	2 (2)	0
Peripheral cyanosis, n (%)	1 (1)		1 (1)	
**Electrocardiogram**	n=156	n=43	n=95	n=28
Frequency, median (IQR), beats/min	76 (65 – 87)	65 (58 – 80)	76 (66 – 88)	64 (58 – 76)
AV block, 1st degree, n (%)	6 (4)		3 (3)	
LBBB, incomplete/ complete, n (%)	0	0	0	0
RBBB, incomplete/ complete, n (%)	6 (4)/ 1 (1)		5 (5)/ 0	
QTc, ms	407 (392 – 425)	405±22	404 (387 – 420)	403±21
T-wave inversion, n (%)	64 (41)	25 (58)	39 (41)	19 (68)
ST elevation, n (%)	76 (49)	20 (47)	53 (56)	16 (57)
ST depression, n (%)	34 (22)	8 (19)	25 (26)	5 (18)
Significant Q-wave, n (%)	7 (4)	5 (12)	5 (5)	3 (11)
Low voltage, n (%)	1 (1)	0		0
Newly reduced R wave height, n (%)	0	1 (2)	0	1 (4)
Other significant finding, n (%) [a]	7 (4)		6 (6)	
**Biochemical parameters**	n=157	n=120	n=94	n=74
Sodium, mmol/l	139 (137 – 141)	140 (139 – 141)	139 (137 – 141)	141 (139 – 142)
Potassium, mmol/l	4.0 (3.8 – 4.2)	4.3±0.4	4.0 (3.8 – 4.2)	4.3±0.3
Ferritin, µg/L	244±173		234±100	
Thyroid stimulating hormone, mIU/L	2.2±1.7	NA	3.5±2.0	NA
Free thyroxine, pmol/L	16 (15 – 19)		19±7	
Total white blood cells (leukocytes), 10^9/L	8.3 (6.3 – 10.4)	6.6 (5.4 – 8.2)	8.4 (6.8 – 10.4)	6.8±2.0
Neutrophile count, 10^9/L	5.7 (3.5 – 7.7)	3.9±2.3	5.8 (3.7 – 8.1)	4.3±3.6
Lymphocyte count, 10^9/L	1.5 (1.1 – 2.1)	2.1 (1.8 – 2.4)	1.4±0.6	2.0±0.3
Platelet count, 10^9/L	219 (179 – 270)	259±71	222±64	250±68
Procalcitonin, ug/L	0.2±0.1	NA	NA	NA
Alanine aminotransferase, U/L	30 (21 – 43)	48±32	32 (22 – 46)	48±34
Aspartate aminotransferase, U/L	38 (21 – 71)	28 (23 – 39)	49 (28 – 83)	25 (22 – 40)
Creatine kinase, U/L	134 (87 – 358)	62 (39 – 886)	157 (57 – 358)	NA
D-dimer, mg/L	0.5 (0.3 – 0.7)	NA	0.5 (0.3 – 0.7)	

Results are reported as median (IQR), n (%), or mean (SD). Data not shown for parameters with limited number of observations (n ≤ 2). Abbreviations: AV, atrioventricular; LBBB, left bundle branch block; NA, not applicable; QTc, corrected QT interval; RBBB, right bundle branch block; VAM, vaccine-associated myocarditis.

[a] Includes left ventricular hypertrophy defined according to Sokolow–Lyon and Cornell criteria (S wave in V1 + R wave in V5 or V6 ≥ 35 mm or R wave in aVL ≥ 11 mm) (n=1), PR-segment depression (n=3), QS configuration in leads V1 and V2 (n=1), and T-wave elevation (n=2).

Supplemental Table 9. Imaging studies at admission of patients with vaccine-associated myocarditis ≤30 days after COVID-19 vaccination

**Chest X-ray, n (%)**	n=71
Pleural effusion	4 (6)
Infiltration	2 (3)
Cardiomegaly	0
Other findings [a]	5 (7)
**Chest CT, n (%)**	n=18
Pulmonary embolus	0
Pneumonia	1 (6)
Pleural effusion	0
Pericardial effusion	1 (6)
Other findings [b]	3 (17)
**Coronary angiography, n (%)**	n=51
CT coronary angiography	28 (55)
Invasive coronary angiography	23 (45)
Atherosclerosis	7 (14)
Stenosis	1 (2)
Calcification<br>Percutaneous intervention [c]	1 (2)
**Echocardiography**	n=41
LV ejection fraction, mean (SD), %	56±4
LV end diastolic diameter, median (IQR), mm	49 (46 – 53)
LV end systolic diameter, median (IQR), mm	37 (29 – 38)
LV end diastolic volume, mean (SD), ml	128±29
Interventricular septum diastolic diameter, mean (SD), mm	9±4
Left atrial volume indexed, mean (SD), ml/m^2	22±12
Global longitudinal strain, mean (SD), %	-19.7±2.6
**Cardiac magnetic resonance**	n=69
LV ejection fraction, mean (SD), %	58±9
LV end diastolic volume indexed, mean (SD), ml/m^2	84±18
LV mass indexed, mean (SD), g/m^2	67±18
Findings consistent with myocarditis, n/N (%) [4, d]	31/43 (72)

Percentages may not total 100% due to missing data or varying response rates. Abbreviations: CT, computed tomography; LV, left ventricular; VAM, vaccine-associated myocarditis.

[a] Includes atelectasis (n=1), pulmonary congestion (n=3), and pulmonary sarcoidosis (n=1).

[b] Includes bilateral lung consolidations without features of infection (n=1), emphysema (n=1), and enlarged lymph nodes (n=1).

[c] Optical coherence tomography–guided balloon dilatation without stent placement.

[d] Defined according to the 2018 revised Lake Louise Criteria, requiring the presence of both T2-based markers of oedema and T1-based markers of non-ischaemic injury. Of the 69 patients with CMR, 43 had both T2- and T1-based sequences acquired and were therefore eligible for full Lake Louise assessment. The percentage is calculated from this subset.

Supplemental Table 10. Additional echocardiographic data at admission and discharge in patients with vaccine-associated myocarditis

Parameter	VAM within 90 days after vaccination		VAM within 30 days after vaccination	
	**Admission (n=72)**	**Discharge (n=13)**	**Admission (n=41)**	**Discharge (n=9)**
LV ejection fraction, %	55 (53 – 60)	55±4	56±4	56±5
LV end diastolic diameter, mm	50±5	49±1	49 (46 – 53)	50±1
LV end systolic diameter, mm	33±5		37 (29 – 38)	NA
LV end diastolic volume, ml	129±26	NA	128±29	NA
LV end systolic volume, ml	50±18		48±21	
Interventricular septum diastolic diameter, mm	9 (8 – 11)	10±2	9±4	9±1
Left atrial volume indexed, ml/m^2	25±11	NA	22±12	NA
Global longitudinal strain, %	-18.1±3.8		-19.7±2.6	
LV posterior wall thickness in diastole, mm	9 (7 – 9)	9±1	9±4	9±1
Pericardial effusion, mm in end-diastole	5±3	5 (5 – 16)	4±2	
E, m/s	0.8±0.2		0.8±0.2	
A, m/s	0.5±0.2	NA	0.5±0.2	NA
Deceleration time, ms	171 (145 – 198)		181±43	
e', cm/s	8.4±5.3		9.3±5.4	
TAPSE, mm	23 (20 – 26)	26±4	23 (20 – 26)	26±4
VCI diameter, mm	17±6	NA	17 (15 – 18)	NA
VCI respiratory variation <50%, n (%)	2 (7)	0	2 (13)	0
TRP, mm Hg	17 (17 – 22)	NA	19±4	NA
Valve disease, n (%)	14 (28)	1 (20)	10 (31)	1 (25)
AR, mild, n (%)	3 (6)		3 (9)	
MR, mild/ moderate/ severe, n (%)	8 (16)/ 1 (2)/ 1 (2)	1 (20)/ 0/ 0	6 (19)/ 0/ 1 (3)	1 (25)/ 0/ 0
TR, mild/ moderate, n (%)	5 (10)/ 1 (2)		4 (13)/ 1 (3)	

Percentages may not total 100% due to missing data or varying response rates. Results are reported as median (IQR), n (%), or mean (SD). Data not shown for parameters with limited number of observations (n ≤ 2).
Abbreviations: AR, aortic regurgitation; LV, left ventricular; MR, mitral regurgitation; NA, not applicable; TAPSE, tricuspid annular plane systolic excursion; TR, tricuspid regurgitation; TRP, tricuspid regurgitation pressure; VAM, vaccine-associated myocarditis; VCI, vena cava inferior.

Supplemental Table 11. Treatment of patients with vaccine-associated myocarditis

Treatment, n (%)	VAM within 90 days after vaccination (n=170)	VAM within 30 days after vaccination (n=102)
Colchicine	68 (40)	50 (49)
NSAIDS	68 (40)	49 (48)
β-blockers for arrhythmia	32 (19)	20 (20)
Heart failure treatment	15 (9)	7 (7)
Systemic steroids	7 (4)	3 (3)
Other anti-inflammatory drugs	4 (2)	2 (2)
Inotropic/vasoactive medication	3 (2)	2 (2)
Other antiarrhythmic drugs	3 (2)	0
Non-invasive respiratory support (CPAP, BiPAP)	2 (1)	1 (1)
Invasive respiratory support	1 (1)	1 (1)

Abbreviations: BiPAP, bilevel positive airway pressure; CPAP, continuous positive airway pressure; NSAIDS, non-steroidal anti-inflammatory drugs; VAM, vaccine-associated myocarditis.

Supplemental References

1. Laugesen K, Ludvigsson JF, Schmidt M, et al. Nordic Health Registry-Based Research: A Review of Health Care Systems and Key Registries. *Clinical Epidemiology* 2021;Volume 13:533-54. doi: 10.2147/clep.s314959
2. Trogstad L, Ung G, Hagerup-Jenssen M, et al. The Norwegian immunisation register – SYSVAK. *Eurosurveillance* 2012;17(16) doi: 10.2807/ese.17.16.20147-en
3. Bakken IJ, Ariansen AMS, Knudsen GP, et al. The Norwegian Patient Registry and the Norwegian Registry for Primary Health Care: Research potential of two nationwide health-care registries. *Scandinavian Journal of Public Health* 2020;48(1):49-55. doi: 10.1177/1403494819859737
4. Ferreira VM, Schulz-Menger J, Holmvang G, et al. Cardiovascular Magnetic Resonance in Nonischemic Myocardial Inflammation. *Journal of the American College of Cardiology* 2018;72(24):3158-76. doi: 10.1016/j.jacc.2018.09.072
