# Supplementary material for: Myocarditis after COVID-19 mRNA vaccination in Norway: a nationwide validation study
Source: Open Heart. 2026 May 4;13(1):e004112. doi: 10.1136/openhrt-2026-004112 (PMC13141113; doi:10.1136/openhrt-2026-004112)
Supplement: online supplemental file 2 [file openhrt-13-1-s003.pdf]

COVID-19 mRNA vaccine-associated myocarditis (VAM) in Norway

Population and methods

- 4.1 million vaccinated individuals
- 10.9 million COVID-19 mRNA vaccine doses

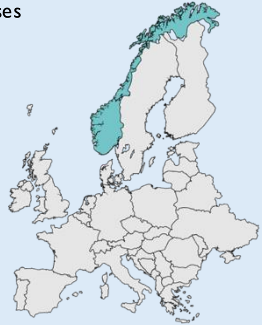

- Myocarditis diagnosis within 90 days after vaccination
- Linkage of national vaccine and diagnosis registries

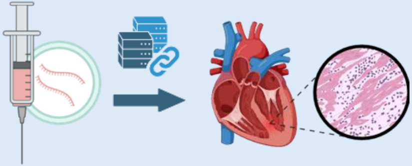

Validation of VAM

367 potential myocarditis cases

349 cases for review

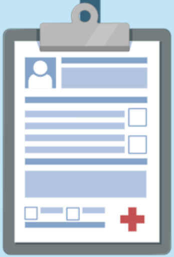

Chart review using Brighton Collaboration criteria

177 validated VAM (51%)

Key findings

VAM was rare:
4.5 VAM per 100 000 vaccinated

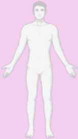

79% (139 cases) men
46% of men aged 18-29 years

62% VAM after second mRNA vaccine dose

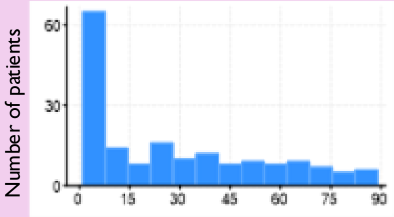

Days from vaccination to VAM

LVEF 55% at admission

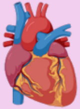

7 (4%) ICU patients

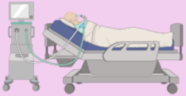

2 (1%) deaths in older adults

Abbreviations: ICU, intensive care unit; LVEF, left ventricular ejection fraction; VAM, vaccine-associated myocarditis
